# Assessing Mechanisms of Potential Local Adaptation Through a Seascape Genomic Approach in a Marine Gastropod, *Littoraria flava*

**DOI:** 10.1093/gbe/evae194

**Published:** 2024-09-05

**Authors:** Thainá Cortez, Gabriel G Sonoda, Camilla A Santos, Sónia Cristina da Silva Andrade

**Affiliations:** Departamento de Genética e Biologia Evolutiva, Instituto de Biociências, Universidade de São Paulo, São Paulo, Brazil; Departamento de Genética e Biologia Evolutiva, Instituto de Biociências, Universidade de São Paulo, São Paulo, Brazil; Laboratório de Toxinologia Aplicada, Instituto Butantan, São Paulo, Brazil; Departamento de Genética e Biologia Evolutiva, Instituto de Biociências, Universidade de São Paulo, São Paulo, Brazil; Tree of Life, Wellcome Sanger Institute, Cambridge, CB10 1SA, UK; Departamento de Genética e Biologia Evolutiva, Instituto de Biociências, Universidade de São Paulo, São Paulo, Brazil

**Keywords:** natural selection, marine invertebrate, transcriptome, non-synonymous and synonymous polymorphisms

## Abstract

Understanding the combined effects of environmental heterogeneity and evolutionary processes on marine populations is a primary goal of seascape genomic approaches. Here, we utilized genomic approaches to identify local adaptation signatures in *Littoraria flava*, a widely distributed marine gastropod in the tropical West Atlantic population. We also performed molecular evolution analyses to investigate potential selective signals across the genome. After obtaining 6,298 and 16,137 single nucleotide polymorphisms derived from genotyping-by-sequencing and RNA sequencing, respectively, 69 from genotyping-by-sequencing (85 specimens) and four from RNA sequencing (40 specimens) candidate single nucleotide polymorphisms were selected and further evaluated. The correlation analyses support different evolutionary pressures over transcribed and non-transcribed regions. Thus, single nucleotide polymorphisms within transcribed regions could account for the genotypic and possibly phenotypic divergences in periwinkles. Our molecular evolution tests based on synonymous and non-synonymous ratio (kN/kS) showed that genotype divergences containing putative adaptive single nucleotide polymorphisms arose mainly from synonymous and/or UTR substitutions rather than polymorphic proteins. The distribution of genotypes across different localities seems to be influenced by marine currents, pH, and temperature variations, suggesting that these factors may impact the species dispersion. The combination of RNA sequencing and genotyping-by-sequencing derived datasets provides a deeper understanding of the molecular mechanisms underlying selective forces responses on distinct genomic regions and could guide further investigations on seascape genomics for non-model species.

**Significance**

The evolutionary mechanisms underlying the distribution of genetic variation and responses to changing environments in marine organisms remain incompletely understood, despite concerning indicators of climate changes. In this study, we employed a widely distributed gastropod species commonly found on tropical Western Atlantic rocky shores and mangroves to investigate this issue. By combining population genomics and transcriptomics data, we discovered distinct selective pressures acting on coding and noncoding regions. Moreover, genetic structuring between localities was observed, driven by significant contributions from both synonymous and noncoding sequence variants. Our research provides valuable insights into the intricate mechanisms governing the response of a non-model species to a continually changing environment.

## Introduction

A more expansive understanding of the connection between genetic structure and local adaptation has become a primary goal in seascape genomic studies (Liggins et al. 2019). This objective can be achieved by combining genomic approaches with techniques capable of detecting associations between environmental factors and the spatial distribution of genomic diversity (Bay and Palumbi 2014; Segovia et al. 2020). By analyzing more loci across the genome, a higher number of regions potentially affected by selection can be accurately identified (Liggins et al. 2019).

Genomic approaches such as RNA sequencing (RNA-Seq) (Gleason and Burton 2015; Huang et al. 2016) and genotyping-by-sequencing (GBS) (Segovia et al. 2020) allow for the identification of thousands of single nucleotide polymorphisms (SNPs) without prior genetic information from the species (Gagnaire et al. 2012; Tepolt and Palumbi 2015). RNA-Seq data also have the advantage of identifying transcribed regions containing the SNPs, which can then be used to predict loci under selection. Additionally, the identification of SNPs under selection in transcript sequences, coupled with open reading frame (ORF) prediction, can be used to assess how particular SNPs impact the amino-acid replacement rate, which is often associated with adaptation (Ingvarsson 2010; Puillandre et al. 2010; Li et al. 2017; Yu and Li 2018).

The complex life histories of many marine invertebrates and the ocean environment's spatiotemporal variability present significant challenges for estimating biogeographic boundaries based on genetic variation measures (Riginos et al. 2016; Liggins et al. 2019). The rocky intertidal shore is one of the most heterogeneous environments known due to the high seasonality of wave action, as well as rapid changes in salinity and temperature occurring over short periods ranging from months to a few hours (Schaal et al. 2011; Shi et al. 2016; Lathlean et al. 2016). Due to the large effective population sizes found in marine invertebrates, this group may be particularly well-suited to respond to environmental changes (Gagnaire et al. 2015) and is often used as an ecological indicator of environmental disturbances in the oceans (Lima et al. 2011; Sousa et al. 2018).

*Littoraria flava* (King 1832), a gastropod widely distributed along the Caribbean and Brazilian rock shores, possesses planktotrophic larvae capable of remaining in the water column for 3 to 6 weeks (Reid 1999). Due to their passive dispersal ability, the larvae can cover vast distances, enabling a high level of admixture even among distant populations along the Brazilian coastline (Andrade et al. 2003; Cortez et al. 2021). Although *L. flava* has high larval dispersal rates, the genetic structure was reported on a microgeographical scale using allozymes and SNPs markers, suggesting the presence of subpopulations within the same rocky shore (Andrade and Solferini 2007; Cortez et al. 2021). In line with these findings, *L. flava* specimens showed differentially expressed genes in specific sites within the same rocky shore, potentially indicating evidence of local adaptation (Santos et al. 2021).

Based on previous studies indicating signs of local adaptation using differentially expressed genes (Santos et al. 2021), we expect to find a significant correlation between candidate SNPs and marine environmental predictors, suggesting the potential existence of unknown biogeographic barriers and selective forces acting at the molecular level. For that, the present study used genomic resources obtained through GBS and RNA-Seq approaches to identify putative adaptive SNPs in populations from the South West Atlantic Ocean. Using these two different datasets, we were able to explore how selective pressures shape genetic diversity in transcribed and presumed untranscribed regions. This integrative approach allowed us to identify signatures in genomic regions with distinct characteristics. After assessing genetic structure and diversity, potential associations between SNPs and marine environmental predictors were evaluated. Additionally, the signatures of potential selective pressures were investigated by predicting the impact of these SNPs on non-synonymous and noncoding substitutions. In doing so, we were able to explore which potential molecular mechanisms are underlying adaptive responses in the species.

## Results

### Datasets

**Fig. 1.**
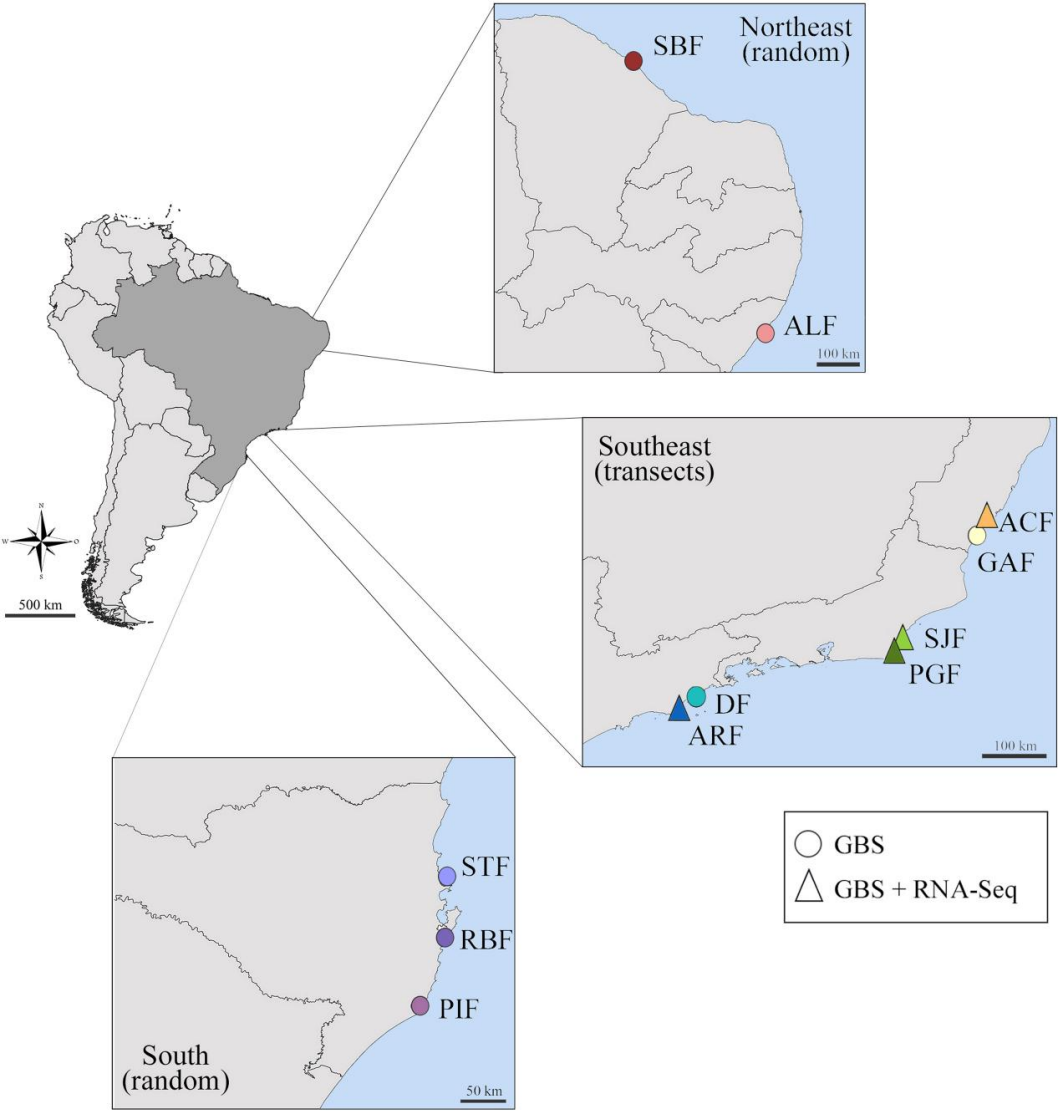
*Littoraria flava* sampling details showing both transects and randomly based collection of animals along the Brazilian coastline. The circles indicate the populations where the individuals were sequenced only through GBS technique, the triangles indicate the populations sequenced by both GBS and RNA-Seq and, therefore, sampled along transects. Abbreviations are indicated in supplementary table S1, Supplementary Material online.

After sampling *L. flava* specimens from 11 locations across the Brazilian coastline (Fig. 1 and supplementary table S1, Supplementary Material online), we obtained 93 specimens for GBS and 40 for RNA-Seq. The library preparation generated 322,479,123 GBS reads. These datasets were originally generated in prior studies to assess both the analysis of differential gene expression through RNA-Seq (Santos et al. 2021) and population structure with GBS (Cortez et al. 2021). After filtering SNPs with minimum allele frequency (MAF) lower than 1% and missing genotypes higher than 20%, 6,298 SNPs from 85 individuals remained (supplementary fig. S1, Supplementary Material online, supplementary table S2, Supplementary Material online). From the RNA-Seq raw data, following methods described in Santos et al. (2021), we obtained 46,863,335 SNPs of 40 different samples. The filtering based on quality (phred score < 30), minimum length of 65 bp, and non-missing data, generated 16,137 SNPs, used in all subsequent analyses 46,863,335 SNPs of 40 different samples (supplementary fig. S1, Supplementary Material online, supplementary table S2, Supplementary Material online). The data used in this study are openly accessible through the SRA database (BioProjects PRJNA665072 and PRJNA656564).

An overview of all datasets used in this study, along with the analyses conducted, is presented in Table 1. Further details for each step are provided below.

**Table 1:** Overview of datasets and corresponding analyses performed in this study

	GBS		RNA-Seq	
	SNPs	Analyses	SNPs	Analyses
Total	6,298	Selection tests (RDA, LFMM, BayeScan)	16,137	Selection tests (RDA, LFMM, BayeScan)
Under selection	2,277	…	506	Multivariate analysis (DAPC, PCA); molecular analysis (kN/kS)
Putative adaptive SNPs	69	Genetic diversity and population structure (θπ, Ho, He, AMOVA, FST); multivariate analysis (DAPC, PCA); functional annotation (BLAST)	4	Functional annotation (BLAST)

For each GBS or RNA-Seq derived data, the number of SNPs is indicated according to the analysis.

Total, number of total SNPs used; under selection, number of SNPs detected as under selection by at least one selection test (RDA, LFMM or BayeScan) applied; putative adaptive SNPs, number of SNPs commonly identified by at least two out of three selection tests, also represented in Fig. 3.

### Putative Candidate SNPs

For both GBS and RNA-Seq, we used two different approaches to identify putative adaptive SNPs: genomic scan based on allele frequencies (BayeScan) and association tests using environmental predictors (latent factor mixed models [LFMMs] and redundancy analysis [RDA]). For the association tests, we first computed the collinearity between each pair of predictors. After removing those with collinearity > 0.8, ten predictors remained: longitude [Long], latitude [Lat], Annual Precipitation [AnnPrec], Precipitation of Wettest Month [PrecWett], Temperature Annual Range [TempAnnRang], Chlorophyll [ChloMean], pH, Salinity, Sea Surface Temperature Range [Sstrange], and Current Velocity Range [Curvssrange] (supplementary fig. S2, Supplementary Material online and supplementary table S3, Supplementary Material online [GBS] and supplementary table S4, Supplementary Material online [RNA-Seq]).

**Fig. 2.**
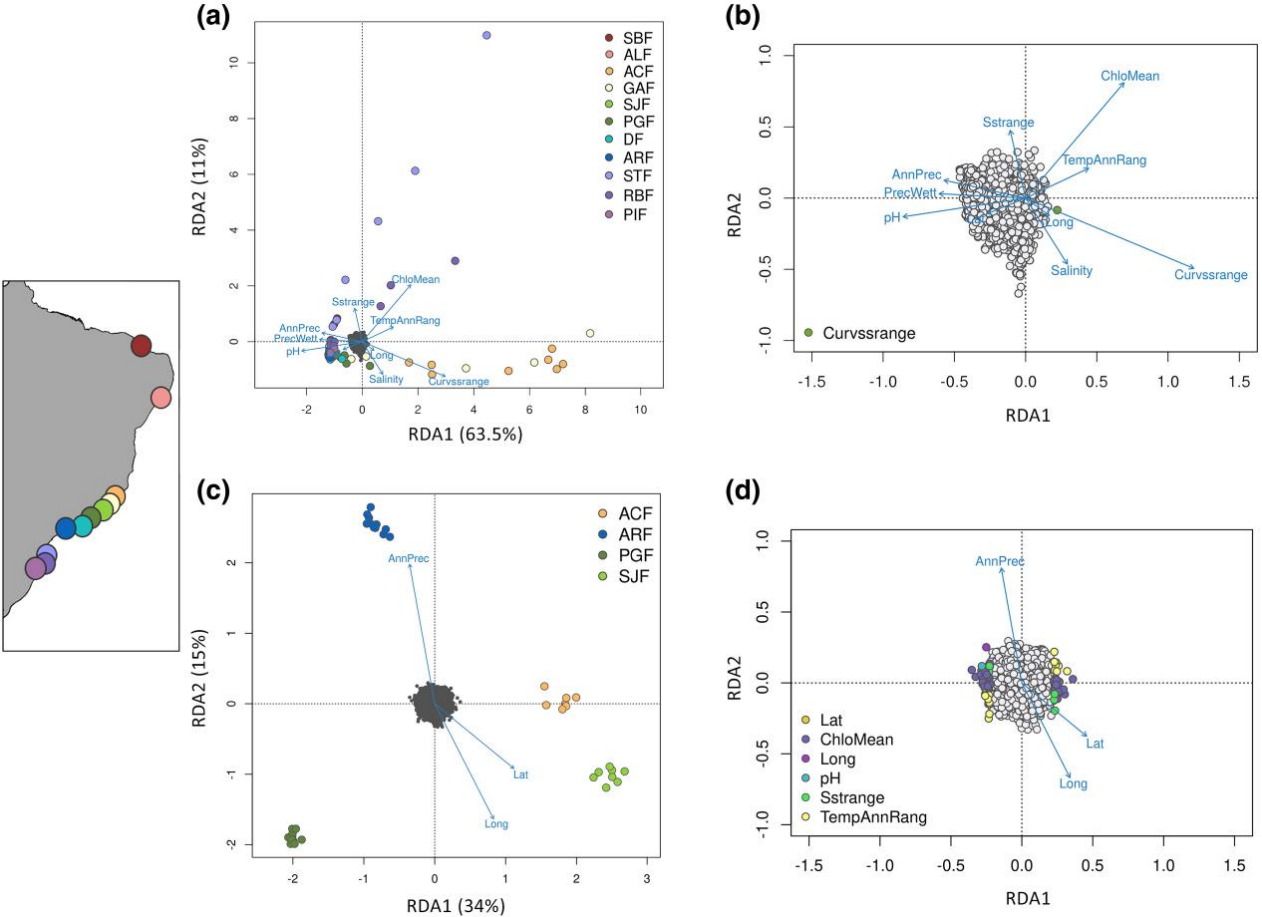
Redundancy analysis (RDA) showing the relative contributions of environmental predictors to the genetic structure of putative adaptive genotypes in *Littoraria flava*. Individual genotypes associations with each predictor are represented for both a) GBS and c) transcriptome candidate SNPs, where SNP are in gray and individuals from distinct locations are represented by different colors according to the map in the left panel. The SNPs significantly associated with each environmental variable are color coded for b) genomic and d) transcriptome dataset (*P* <0.01). For GBS (a and b), individuals from STF, ACF and GAF are separated from the remaining on the first PC; while for transcriptome (c and d), individuals are geographically separated.

For those 6,298 GBS-derived SNPs, the BayeScan showed that 78 SNPs within 60 loci are potentially under selection (false discovery rate [FDR] < 0.05) (supplementary fig. S3, Supplementary Material online). The RDA full model based on 6,298 SNPs and all ten predictors was significant (*P* < 0.01) (input as supplementary Appendix S1, Supplementary Material online) showed a correlation of 15% between the environmental and genomic variation. According to the relative arrangement of the samples in the ordination space of axis 1 (*P* = 0.01), the individual genotypes from Espírito Santo samples (ACF and GAF) might be positively related to the current velocity range (Curvssrange), meaning that individuals from Espírito Santo are under higher current velocity ranges over the year (Fig. 2a). However, these populations present the highest missing data values and, therefore, such outliers might result from differences in genotypes representation.

**Fig. 3.**
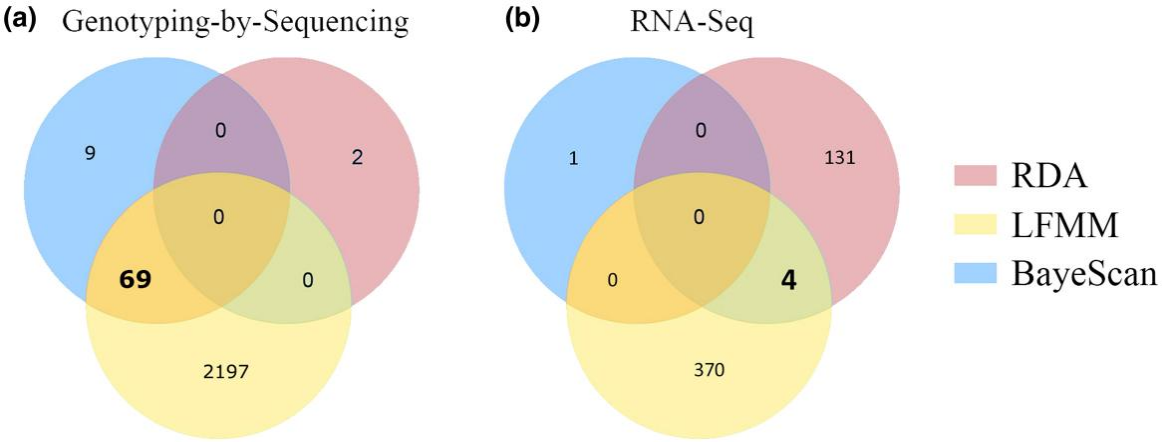
Venn diagram of the intersection of SNPs containing candidate SNPs identified by RDA, LFMM, and BayeScan for a) GBS dataset of *Littoraria flava* and b) RNA-Seq. In bold, the final subset of candidate SNPs used for the subsequent analyses.

Two candidate SNPs from different loci were associated with Curvssrange (Fig. 2b). The LFMM revealed that 2,266 SNPs were associated with at least one environmental predictor (Fig. 3a). The predictors with the most correlated SNPs were Curvssrange (1,009) followed by pH (830), with 406 SNPs shared between these two. The mapping of all GBS loci containing candidate SNPs resulted in 46 mapped loci (∼5% of 6,298), where about 60% showed the first hits with the gastropod *Pomacea canaliculata* proteins, which correspond to a pleiotropic regulator, ubiquitin-protein ligase, calcineurin-binding protein, and zinc finger protein (supplementary table S5, Supplementary Material online).

For the RNA-derived dataset, the BayeScan algorithm identified one single candidate SNP (supplementary fig. S4, Supplementary Material online and Fig. 3b) without any annotation available. The full RDA model based on 16,137 SNPs (input as supplementary Appendix S2, Supplementary Material online) was also significant (*P* < 0.01) and showed a correlation of 0.2% between the environmental predictors and the genomic variation. A slightly positive relationship of samples from the São João and Anchieta with higher latitude was detected in the first axis (*P* < 0.01) (Fig. 2c). The sample distribution showed complete segregation of individual genotypes from different locations across the first axis. Also, 135 SNPs within 128 loci were associated with environmental predictors: eight with pH, 31 with Sstrange, 59 with ChloMean, one with Lat, 30 with TempAnnRang, and six with Long (Fig. 2d). None shared two or more predictors. The LFMM analysis detected 374 SNPs associated with at least one environmental predictor, with AnnPrec and Long exhibiting the most associated variants (159 and 150, respectively). Additionally, pH and Curvssrange were associated with 88 and 114 SNPs, respectively. Overall, only four variants were commonly identified by RDA and LFMM (Fig. 3b), which seem related to tRNA ligases and apoptosis (supplementary table S6, Supplementary Material online).

After the adaptive SNPs identification, we looked into functional aspects of those variants. For that, we set the threshold that only variants identified in two of the three analyses would be investigated in terms of potential biological functions. Therefore, the final number was 69 and four candidate SNPs for GBS and transcriptome, respectively (Fig. 3).

### Population Diversity and Structure

**Fig. 4.**
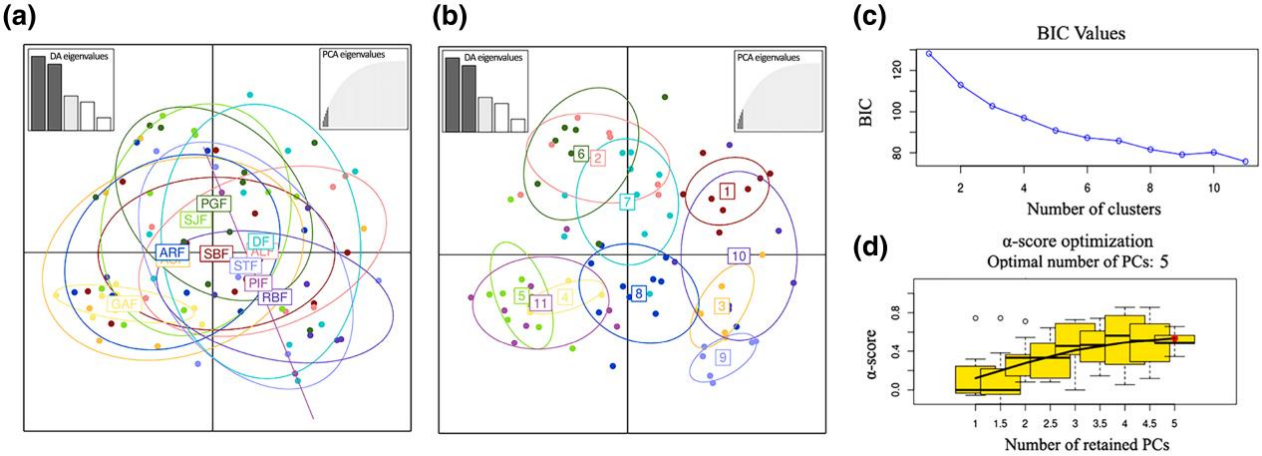
Discriminant analysis of principal components (DAPC) performed based on 69 GBS candidate SNPs from 85 individuals of *Littoraria flava*. The distribution of individuals across the two first principal components (PCs) colored according to a) the sampling locations and b) the genetic clusters (K = 11) according to the c) lowest BIC from DAPC, showing that the specimens’ distribution lacks any geographic pattern. Each dot represents an individual. Both DAPCs were performed using five PCs, as suggested by the d) α-score optimization.

The measures of genetic diversity and allele polymorphism of the 69 GBS candidate SNPs are presented in Table 2. The θπ values ranged from 0.77 to 7.18 in Gamboa (GAF) and Praia Dura (DF), respectively. The difference between the expected and observed heterozygosity means was significant (*P* < 0.05), along with a significant and relatively high *F*_IS_ index (average = 0.606, *P* < 0.05). When considering all individuals within the same region (Northeast [NE], Southeast [SE], or South [S], see Table 2), the NE exhibited higher diversity despite smaller sampling size. On the other hand, the SE presented the lowest nucleotide diversity, even with the largest sample size. The AMOVA results revealed that most genetic variability (∼63%) occurs within locations (supplementary table S7, Supplementary Material online). The global *F*_ST_ (*P* = 0.892) and all the pairwise *F*_ST_ values (*P* > 0.05) failed to reach a level of significance. The discriminant analysis of principal components (DAPC) analysis based on five principal components (PCs), as suggested by the *optim.a.score*, revealed 11 genetic clusters, which did not present any clear geographic pattern, even when colored according to the regions of Brazilian coastline (Fig. 4 and supplementary fig. S5, Supplementary Material online).

**Table 2:** *Littoraria flava* genetic diversity indexes based on the 69 GBS candidate SNPs

Location	Samples (*n*)	θπ	H_O_	H_E_	*F*_IS_
Northeast (NE)					
Region	17	1.7	0.082**	0.341	0.319
SBF	11	2.47	0.106**	0.412	0.166
ALF	6	5.66	0.120**	0.405	0.615
Southeast (SE)					
Region	53	0.628	0.147**	0.339	0.538*
ACF	8	1.45	0.125	0.362	1.00**
GAF	8	0.77	0.125	0.258	0.00
SJF	10	5	0.078**	0.357	1.00*
PGF	10	3.27	0.150**	0.327	0.640
DF	9	7.18	0.163**	0.422	−0.23
ARF	8	3.61	0.125*	0.301	0.740
South (S)					
Region	15	0.646	0.066	0.215	1.00*
STF	6	2.21	0.055*	0.369	1.00
RBF	7	1.16	0.143	0.291	1.00*
PIF	2	5	0.556	0.556	NA

Samples (*n*)—sampling size; θπ—nucleotide diversity; HO and HE—observed and expected heterozygosity, respectively. The values are shown for each location and for the regions, i.e. all populations from the same region as one single population unit. Abbreviations as in Fig. 1 and supplementary table S1, Supplementary Material online.

*P < 0.05.

**P < 0.01.

### Synonymous and Non-Synonymous SNPs

**Fig. 5.**
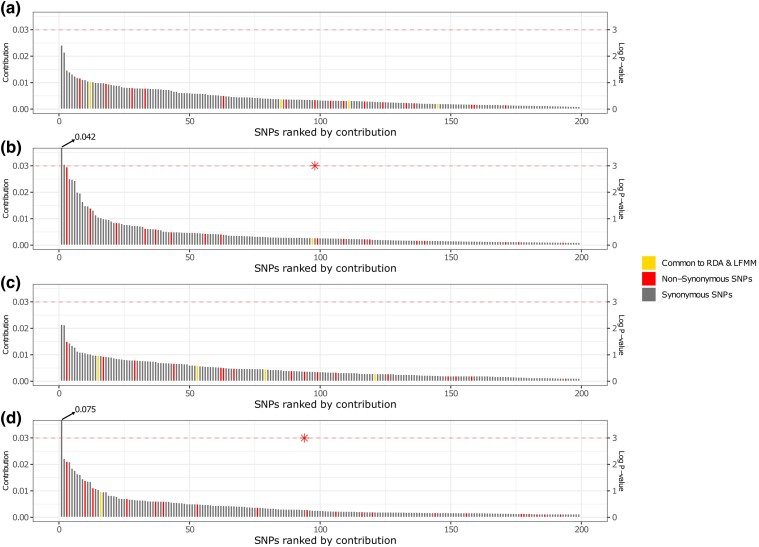
Contribution of the top 200 SNPs in the descending contribution rank to the discriminant functions 1 a) and 2 b) of discriminant analysis of principal components (DAPC) and principal components 1 c) and 2 d) of principal components analysis (PCA). Each bar corresponds to the percentage of contribution of the SNP to the component analysis axis. The yellow bars represent the SNPs identified by RDA and LFMM, all of which were synonymous SNPs. At the first iteration, the top ten SNPs from the descending rank of were tested and, with each iteration, the following SNP of the rank is added to the test. The dashed red line represents the *P*-value log (0.05). Red asterisks are *P* < 0.05.

To assess the selection forces acting on the translated amino-acid sequences, we compared the amount of non-synonymous (kN), synonymous (kS) SNPs, and outside coding sequence (oCDS) within neutral and putative adaptive SNPs from the transcriptome dataset only. From all of the 506 adaptive SNPs derived from the transcriptome (according to at least one of the selection analyses, Fig. 3), 303 were annotated within CDS. Of these, 266 were synonymous, and 37 were non-synonymous, resulting in a kN/kS = 0.139 (supplementary table S8, Supplementary Material online). The kN/kS ratio in the 9,287 neutral SNPs annotated within CDS was 0.148 (supplementary table S8, Supplementary Material online). Pearson's Chi-square test showed no difference in the kN/kS proportion between the set of putatively adaptive SNPs and the putatively neutral ones (*P* > 0.7). Also, none of the four SNPs commonly found by RDA and LFFM (putative candidate SNPs section) were non-synonymous changes (Fig. 5).

Subsequently, we described the differences in adaptive SNP frequency between localities by applying both principal component analysis (PCA) and DAPC from R packages “FactorMineR” and “adegenet”. We also evaluated the contribution of kN SNPs to the obtained principal components. These analyses revealed that it was possible to ascertain the role of substitutions affecting the translated amino-acid sequence on local selection. The first and second axis of the PCA represented 56.9% and 27.1% of the variance, respectively (supplementary fig. S6, Supplementary Material online), totaling 84% of all variation within the data. The DAPC was performed with six PCs as suggested by the function *optimal.a.score* (supplementary fig. S6, Supplementary Material online). The eigenvalues of the first and second discriminant functions, interpreted as the variance ratio between the defined cluster to the variance within these clusters, were 181.2 and 8.4, indicating a crucial role of the first component in segregating the localities. Both the PCA and DAPC resulted in the first component separating AR and DF from SJF and ACF. Moreover, the second axis separates ACF from SJF (supplementary fig. S6, Supplementary Material online). Neither the first PCA component nor the first DAPC discriminant function displayed a significant excess of kN SNPs at the top of the contribution ranking as confirmed by the Wilcoxon rank sum test (Fig. 5). Conversely, a nonrandom (*P* < 0.05) distribution of kN, kS, and oCDS SNPs in the contribution rank was only observed when testing the top 94 and 98 SNPs from the second principal component and second discriminant function (Fig. 5).

## Discussion

We combined genome and transcriptome-wide SNPs obtained from 125 specimens with seascape attributes to detect potential local adaptation in a highly dispersive marine invertebrate. While previous neutral genetic structures indicated the presence of three genetic clusters (Cortez et al. 2021), we found 11 putative adaptive genetic clusters without any clear geographic pattern. In addition, we observed different segregation patterns across localities for adaptive SNPs derived from GBS and RNA-Seq, revealing that transcribed and supposedly non-transcribed regions seem to be experiencing distinct selective forces. Remarkably, non-synonymous substitutions did not contribute significantly within the transcribed regions, in contrast to the highly relevant role of synonymous and oCDS substitutions for genetic structure.

Here, the implemented methods showed good performance for both datasets. Despite the difference in the number of identified SNPs, we were able to recover SNPs commonly found in at least two out of the three analyses, reducing the false positives and providing a more robust dataset. For the association tests, even though public databases do not yet encompass all conditions capable of predicting genetic diversity, the currently available seascape attributes have provided relevant information about biogeographic barriers and selective forces in the marine realm (Riginos et al. 2016), particularly when integrated with high-throughput sequencing technologies (Galindo et al. 2010; Westram et al. 2014). We have found divergent results with RNA and GBS-derived SNPs, suggesting different independent selective pressures acting in transcribed and supposedly non-transcribed genomic regions (Pespeni et al. 2012). Additionally, only transcriptome candidate SNPs showed a clear geographic distribution pattern of genotypes in the RDA (Fig. 2c), probably reflecting environmental differences, such as human activities, in these areas (Sterza and Fernandes 2006). Nonetheless, the distribution of individual genotypes in RDA ordination can also be influenced by missing data rate. For example, populations ACF and GAF show higher rates of missing data in the GBS dataset, and their specimens are depicted sparsed in the RDA plot (Fig. 2). The pattern observed could be related to the missing data, so these populations’ results are interpreted carefully.

Eleven adaptive genetic clusters were found when analyzing putative adaptive SNPs from GBS, despite lacking any geographic pattern (Fig. 4). Previous findings with putatively neutral SNPs showed three discrete genetic clusters from the same collection locations, presented in Cortez et al. (2021). Even presenting a high level of admixture, the marine currents seem to be able to shape the distribution of genomic variation of *L. flava* and other invertebrates (Benestan et al. 2016; Miller et al. 2019). The candidate SNPs still showed a nonsignificant *F*_ST_ and high nucleotide diversity. Also, the diversity indices were higher for populations in Northeastern and Southern localities, despite the smallest sampling size when considering both locations or regions as a population unit (Table 2). For the Southeastern localities, besides the excess of heterozygotes for some local populations, a higher admixture level or a common origin of the individual genotypes is supported. Most genomic variation is mainly explained by individuals within the rocky shores instead of in large-scale coastal regions. Together with the AMOVA and *F*_ST_ results from neutral SNPs (Cortez et al. 2021), these findings keep supporting highly connected *L. flava* populations across the Brazilian rocky shore, even though unclear sources of selective forces might affect the genetic diversity distribution. Thus, although the current velocity range was the only predictor commonly found for the GBS and transcriptome candidate SNPs, other predictors, including pH, water surface temperature, chlorophyll, precipitation and temperature range, influence the distribution of transcriptome-derived SNPs. These conditions are known to be relevant for the performance and survival of species that inhabit rocky shores and are exposed to both marine and terrestrial conditions (Harley and Helmuth 2003; Fuchs et al. 2010; Benestan et al. 2016; Miller et al. 2019; Sauerland et al. 2019; Takeuchi et al. 2020; Mendes et al. 2022).

From RNA-Seq candidate SNPs, two loci gene products are related to apoptosis: serine/threonine-protein phosphatase 6 (PP6) and apoptosis-inducing factor 1 (AIF1) (supplementary table S6, Supplementary Material online). AIF1 was also identified and characterized in *Crassostrea gigas* by Qiao et al. (2021) as participating in apoptosis triggered by immune responses. Apoptosis may be triggered during immune responses caused by environmental disturbances, such as pollution, hormones, temperature, and pH variations (Romero et al. 2015; Qiao et al. 2021). In gastropods, cell death has also been reported to follow cellular damage after exposure to heavy metals and xenobiotics (Chabicovsky et al. 2004; Russo and Madec 2007). A PP6 loci variant was identified as under selection, which was also found up-regulated in São João gastropods (Santos et al. 2021), an area known for fishing, boat activities, and high levels of pollutants in the water, which may lead to a stressful scenario for fauna living along rocky shores (Oigman-Pszczol and Creed 2007; do Nascimento et al. 2018). Interestingly, PP6 is believed to be involved in shell formation and biomineralization processes and ocean acidification changes can represent a higher vulnerability for gastropods larvae development or calcification (Kurihara 2008; Ellis et al. 2009; Gazeau et al. 2010; Duarte et al. 2013; Rajan and Vengatesen 2020).

We investigated the impact of local selection on gene variation. For this purpose, transcriptomic data allowed us to compare the contribution of kN SNPs, kS SNPs, and oCDS SNPs to potential local adaptation. We found that the ratio of kN/kS was similar between putatively adaptive and the whole set of SNPs (supplementary table S8, Supplementary Material online). This similarity suggests these variants may play equivalent roles or have no adaptive significance. Accordingly, the Wilcoxon rank sum tests for the first component of both the PCA and DAPC, which segregates ARF and PGF from SJF and ACF (supplementary fig. S6, Supplementary Material online), suggests that genetic clustering between these localities did not arise mainly from the natural selection of polymorphic proteins, but from significant contributions of synonymous SNPs and oCDS SNPs. On the other hand, Wilcoxon rank sum tests indicates that kN SNPs are not randomly distributed through the contribution rank for the second axis of PCA and DAPC (*P* < 0.05) (Fig. 5), both of which segregate SJF from ACF (supplementary fig. S6, Supplementary Material online). The eight kN SNPs present in the significant Wilcoxon tests have a high contribution to the segregation of the aforementioned localities, which are close to each other. The nonrandom ranking of kN SNPs contribution is suggestive of amino-acid level divergences between localities, but there is an enormous contribution of kS SNPs and oCDS SNPs for all components. Further nonrandom distribution of kN SNPs in the contribution rank can hardly be confirmed by visual inspection of the data (Fig. 5).

Through mechanisms previously suggested, such as degradation rates and splicing efficiency of the mRNA (Chamary et al. 2006; Liu et al. 2021), these SNPs might be affecting the expression patterns between localities as a result of local selection. Synonymous SNPs under selection affecting translation efficiency were detected in human evolution (Waldman et al. 2011). An alternative hypothesis is that these SNPs are not directly selected but are genetically linked to polymorphic promoters, usually close to their respective transcript in eukaryotic genomes (Haberle and Stark 2018). Both hypotheses could participate in the differentiation of gene expression across localities, a pattern observed for *L. flava* (Santos et al. 2021). However, linkage between kS and kN SNPs present in unsampled transcripts should not be discarded. (*P* < 0.05)

Our findings suggest that amino-acid polymorphisms (resulting from kN SNPs) play a minor role in local selection for *L. flava* adults. SNPs selected because of different ecological conditions were mostly kS and oCDS. Presence of putatively adaptive kS SNPs was previously observed in oysters from the genus *Crassostrea* (Zhong et al. 2014) and in fish from the species *Merluccius merluccius* (Milano et al. 2014), and positively selected sites in UTRs were found in *Drosophila* species (Haddrill et al. 2008). The contribution of adaptive oCDS SNPs to genetic structure was discussed for populations of sea urchins, although these were in regions upstream to the transcription start site, not in UTRs, as in our results (Pespeni et al. 2012). In line with that, reduction of spines in threespine sticklebacks provides an example of adaptive genetic variation in untranscribed regions (Shapiro et al. 2004; Chan et al. 2010). Despite the presented evidence for kS and oCDS SNPs participation in local selection, some authors treat kS SNPs as false positives instead of considering the possibility of selection through other means (Cullingham et al. 2014). Combined with previous researches, our results suggest that these SNPs should not be overlooked as they represent a substantial portion of the polymorphisms putatively under selection.

Our study presents an attempt to understand the role of kS and transcripted oCDS SNPs in local selection in a non-model organism. These findings have great potential for future local adaptation studies regarding the mechanisms driving adaptive evolution in natural populations. Furthermore, implementing molecular research alongside environmental association tests highlights the benefits of integrative approaches for understanding natural selection dynamics, especially for the complex marine realm.

## Materials and Methods

### Biological Sampling

We collected *L. flava* specimens from rocky shores at 11 locations along the Brazilian coastline (supplementary table S1, Supplementary Material online) under the Instituto Chico Mendes de Conservação da Biodiversidade license no. 56726-1. In four of these localities (Araçá, Praia Dura, São João, and Anchieta), samples were collected along horizontal transects to examine the effect of microhabitat distribution on SNP frequencies and gene expression (Fig. 1). The transect on the rocky shore extended from the furthest point from the sea to the closest point to the sea where the species was present, varying from 0 to 64 m of distance. Sampling methodology details can be found in Cortez et al. (2021) and Santos et al. (2021). Whole-body individuals were then separately subjected to RNA and DNA extraction.

### GBS and SNPs Call

We extracted genomic DNA following Doyle and Doyle's (1987) protocol and quantified DNA concentrations using a dsDNA BR Assay kit (Invitrogen, Waltham, MA, USA) with a Qubit v3 fluorometer (ThermoFisher Scientific, Waltham, MA, USA). Libraries were constructed based on the GBS method following the protocol of Elshire et al. (2011) and using the *Pst*I restriction enzyme (5′-CTGCAG-3′). Once ligated to the barcode and common adaptors, the products were PCR amplified using generic primers matching the common adaptors under the following conditions: 5 min at 72 °C, 30 s at 98 °C, 18 cycles of 10 s at 98 °C, 30 s at 65 °C, and 30 s at 72 °C, and an extension step of 5 min at 72 °C. The size and quality of the DNA fragments were confirmed using an Agilent 2100 Bioanalyzer (Agilent Technologies, Santa Clara, CA, USA) with the Agilent DNA 1000 kit and by qPCR on a Light Cycler 480II (Roche). The Kapa Biosystems kit was used for library quantitation. The GBS libraries were constructed at the EcoMol Consultoria (Piracicaba, SP, Brazil) and sequenced at the Center for Functional Genomics Applied to Agriculture and Agroenergy (Animal Biotechnology Laboratory, LZT/ESALQ/USP, Piracicaba, SP, Brazil) on a HiSeq 2500 platform (Illumina Inc., San Diego, CA, USA).

The quality of GBS raw reads was checked with FastQC v.0.11.8 (Andrews 2010). Seqyclean v.1.9.9 (Zhbannikov et al. 2017) through the UniVec database (NCBI, ftp://ftp.ncbi.nlm.nih.gov/pub/UniVec/) was utilized to remove adapter sequences, vectors, oligonucleotides, and reads with average Phred (QS) quality below 24 and smaller than 50 bp. Individual read assignments, paralog identification, and read clustering into consensus sequence for each locus were performed with the iPyrad v.0.7.28 program (Eaton 2014). All program-specific input formats were obtained with PGDSpider v.2.1.15 (Lischer and Excoffier 2012). PLINK v.2.0 (Purcell et al. 2007) was used to remove SNPs with a MAF of <0.01 and missing genotypes per SNP of >0.20. Details on the filtering results can be found in Cortez et al. (2021).

### RNA-Seq, Transcriptome Assembly, and SNPs Call

We isolated the RNA from entire *L. flava* animals obtained at each site using Trizol, according to Riesgo et al. (2012). The RNA quantity was verified using the Qubit fluorometer and NanoDrop spectrophotometer (ThermoFisher Scientific). The integrity of the samples was confirmed using BioAnalyzer (Agilent Technologies Inc.), and only samples with RNA integrity number (RIN) values of >6.0 were selected for subsequent analysis. A total of 40 libraries were constructed and sequenced as described in Santos et al. (2021).

After sequencing, we visualized the quality of the raw data generated with FastQC software. All the reads for adapters, primers, and quality were filtered using SeqyClean with QS with the mean and edge minimum score values set at 24 and 30, respectively, and a minimum length of 65 bp. We performed a de novo assembly using Trinity v.2.8.4 (Grabherr et al. 2011), as described in Santos et al. (2021) and used the TransDecoder package (http://transdecoder.sourceforge.net/) to identify the candidate coding regions. Next, the functional annotation was performed using the Trinotate pipeline (https://trinotate.github.io/) with BLASTx in the following databases: UniProt (uniref90 + SwissProt) with a cutoff value of 1e10-3; Gene Ontology (GO) (Ashburner et al. 2000), with GO terms: biological process (BP), molecular function (MF), and cellular component (CC); and KEGG (Kyoto Encyclopedia of Genes and Genomes) (Kanehisa et al. 2012).

We then mapped the reads of the 40 *L. flava* samples against the reference unigenes with BLASTx hits using Bowtie2 v.2.3.4.3 (Langmead and Salzberg 2012). Using the mpileup command of Samtools v.1.3 (Li et al. 2009), we detected SNPs on annotated transcripts using the parameters -q20, -C50, -A (do not discard anomalous read pairs) and -B (disable base alignment quality inference). With BCFtools v.1.3 (Li et al. 2009) for SNP calling, we excluded variant base quality of <30 and the sum of the coverage of reads with alternative alleles in the forward and reverse (DP4) strands of <10 from the analyses to avoid artifacts. We only considered variants present in at least four individuals (10%), with a minimum frequency of 98% and MAF < 0.01 for downstream analysis, using the options –mac 4, --max-missing 0.98 and --maf 0.01in vcftools v.0.1.16 (Danecek et al. 2011).

### Putative Candidate SNPs

We conducted a genome scan and two environmental association tests to identify the best candidate SNPs for local adaptation using RNA-Seq and GBS read datasets. By employing the Bayesian approach from BayeScan v.2.0 (Foll and Gaggiotti 2008), we used a genome scan to detect deviations from neutrality. We set a prior odd of 10, which assumes that the neutral model is ten times more likely than the selection model. The program ran 50,000 iterations followed by 500,000 simulations, with a burn-in of 1% and a FDR of 5%.

We performed environmental association tests using RDA (Forester et al. 2018) and LFMM (Frichot and François 2015). Previous studies have suggested that several environmental variables, such as salinity (She et al. 2018), sea temperature (Dwane et al. 2023), and dissolved oxygen (San et al. 2021), can drive local adaptation in marine invertebrate species. Therefore, we downloaded continental and oceanic environmental data from sampling locations from WorldClim (www.worldclim.org) and Bio-ORACLE databases (Tyberghein et al. 2012). We extracted data for each locality using the *sdmpredictors* library (Bosch et al. 2017) from the statistical software R v.3.6 (R Core Team 2013). We tested the collinearity of data using RDA from the package “vegan” 2.6.2 (Oksanen et al. 2013). We removed all values greater than 0.8, retaining only ten predictors (Long, Lat, AnnPrec, PrecWett, TempAnnRang, ChloMean, pH, Salinity, Sstrange, Curvssrange; see Results section). We tested the significance of the full model and each constrained axis using an analysis of variance with 1,000 permutations. We applied a standard deviation cutoff of ±3 from the mean SNP loading for each axis (Forester et al. 2018).

We implemented the LFMM test using R package “LEA” 3.15 (Frichot and François 2015) for the same ten environmental predictors used in RDA. We tested each predictor for each K using a burn-in of 2,000 followed by 20,000 iterations, setting the number of latent factors according to the minimal cross-entropy criterion of the *snmf* function (K varying from 1 to 20). We adjusted the *P*-values based on the median z-score (Storfer et al. 2018) and adopted a significance level of 1% (*P* < 0.01). We defined the set of candidate SNPs for local adaptation as those found in at least two of the three analyses (i.e. BayeScan, RDA, and LFMM) for both datasets, which are hereafter referred to as GBS and transcriptome candidate SNPs.

The GBS candidate SNPs were mapped against the previously annotated transcriptome of *L. flava* using Bowtie2 v.2.4.4 (Langmead and Salzberg 2012). Functional annotation for transcriptome candidate markers was performed with the BLASTn Search tool from BLAST v.2.9.0 (Camacho et al. 2009) using the nucleotide collection and *e*-value threshold of 10^−3^.

### Genetic Diversity and Structure for Candidate SNPs

For the GBS candidate SNPs, we calculated multi-locus expected heterozygosity (H_E_), observed heterozygosity (H_O_), nucleotide diversity (θ_π_), and the fixation index *F*_IS_ (Weir and Cockerham 1984) for the GBS candidate SNPs using Arlequin v.3.5 (Excoffier and Lischer 2010). The Bartlett test was applied for the variance of observed and expected heterozygosity and obtained the significance of the mean differences using the paired *t*-test. We tested the significance level with 10,000 permutations (*P* < 0.05). The genetic differentiation was evaluated using the AMOVA from Arlequin, considering (i) each locality and (ii) each region (NE, SE, and S, see supplementary table S1, Supplementary Material online), based on the unbiased *F*_ST_ estimator θ (Weir and Cockerham 1984).

We used DAPC (Jombart et al. 2010) as an assumption-free genetic clustering method to assess adaptive genetic structure. This approach was preferred since the genetic clustering algorithm from STRUCTURE (Pritchard et al. 2000) requires Hardy–Weinberg equilibrium and lack of linkage disequilibrium in ancestral populations (Frichot et al. 2014). We performed the DAPC using the optimal number of PCs (*n* = 5, see results) suggested by the optim.a.score function from the “adegenet” R package (Jombart 2008) and defined genetic clusters as the locality of the individuals using the *find.cluster* function.

### Synonymous and Non-Synonymous SNPs Analyses

To understand how local selection affects SNP frequency in transcribed sequences, we annotated and classified all filtered SNPs detected in the RNA-Seq data as synonymous, non-synonymous, or oCDS using SnpEff v.5.0 (Cingolani et al. 2012) with the candidate CDS previously identified by TransDecoder as a reference.

We filtered out transcripts containing more than one predicted ORF since these could result in ambiguously annotated SNPs. As only 46 GBS SNPs putatively under selection could be mapped to transcribed regions, these SNPs were not considered here. The number of SNPs was counted and classified as non-synonymous (kN) (except for loss of sense), synonymous (kS), and untranslated regions (i.e. oCDS) for both neutral and putatively adaptive SNPs. For that, we used a custom bash script (supplementary Appendix S3, Supplementary Material online) and a variant calling file (vcf) generated by SnpEff. We defined neutral SNPs as those identified by none of the analyses (BayeScan, RDA, and LFMM). As too few SNPs were found by at least two predictors, for this analysis, we considered putatively adaptive SNPs as those 506 SNPs identified by at least one of the three analyses. Pearson's Chi-squared test using the R package “stats” (R Core Team 2013) was performed to assess the similarity in the proportion of kN and kS SNPs between the two sets.

To describe the contribution to local selection provided by SNPs that change the translated amino-acid chain (kN) and those that do not (kS and oCDS), we used principal component approaches. We performed a PCA using the “FactoMineR” (Lê et al. 2008) package and a DAPC using the “adegenet” package. The PCA was calculated using each locality as an observation and bi-allelic amino-acid polymorphism frequencies as variables. We set each locality as a genetic cluster for DAPC (PCs = six, according to the optim.a.score function). The PCA and DAPC components segregated individuals from different localities based on SNPs’ frequency variance. We expect that variation in SNP frequencies across different localities may result from distinct selective forces reflecting the particularities of the biotic and abiotic conditions. These dynamics could, then, lead to local adaptation.

Afterward, we compared the contribution of kN SNPs to the contribution of kS and oCDS SNPs (previously annotated by snpEff) in the first two components of the PCA and DAPC. For that, we ranked the SNPs by their contribution to each component in descending order and submitted the top 200 SNPs (∼90% of total contribution) of each component to a one-tailed Wilcoxon rank sum test (Bauer 1972). We compared the ranks of kN SNPs to the ranks of kS and oCDS SNPs. With this test, we assessed if kN SNPs are randomly distributed through the contribution rank or if they are more prone to be at the top. We used a custom R script (supplementary Appendix S4, Supplementary Material online) that starts with the ten top-ranked SNPs and successively adds the following SNPs in the descending contribution, applying a one-tailed Wilcoxon rank sum test at each iteration, until the contribution of the top 200 SNPs was tested. The iterations increase the sensibility by removing SNPs with a negligible contribution to the components, which could add noise to the analyses.

## Supplementary Material

evae194_Supplementary_Data

## Data Availability

RNA-Seq and GBS-derived raw sequence reads are deposited in the SRA (BioProjects PRJNA665072 and PRJNA656564, respectively).
