# Supplementary material for: Assessing Mechanisms of Potential Local Adaptation Through a Seascape Genomic Approach in a Marine Gastropod, Littoraria flava
Source: Genome Biol Evol. 2024 Sep 5;16(9):evae194. doi: 10.1093/gbe/evae194 (PMC11413584; doi:10.1093/gbe/evae194)
Supplement: evae194_Supplementary_Data [file evae194_supplementary_data.zip › Supplementary_information_Figures.docx]

**Assessing mechanisms of local adaptation through a seascape genomic approach in a marine gastropod, *Littoraria flava***

Thainá Cortez, Gabriel G. Sonoda, Camilla A. Santos, Sónia C. S. Andrade

# Supplementary Figures


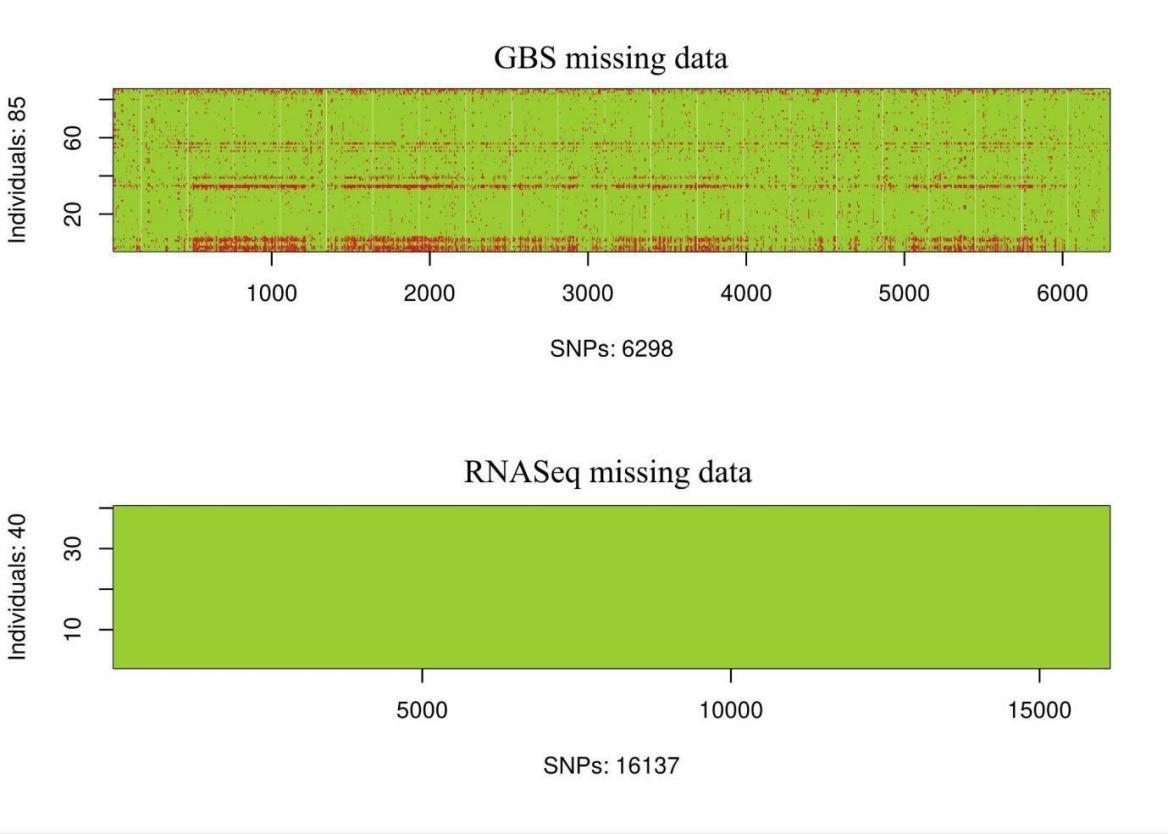


**Fig. S1.** Occupancy matrix for both GBS (n = 85) and RNASeq (n = 40) datasets of *Littoraria flava* showing the distribution of missing data per sequenced specimen. Each red dot represents a single missing data for the specific variant (SNP) and individual.


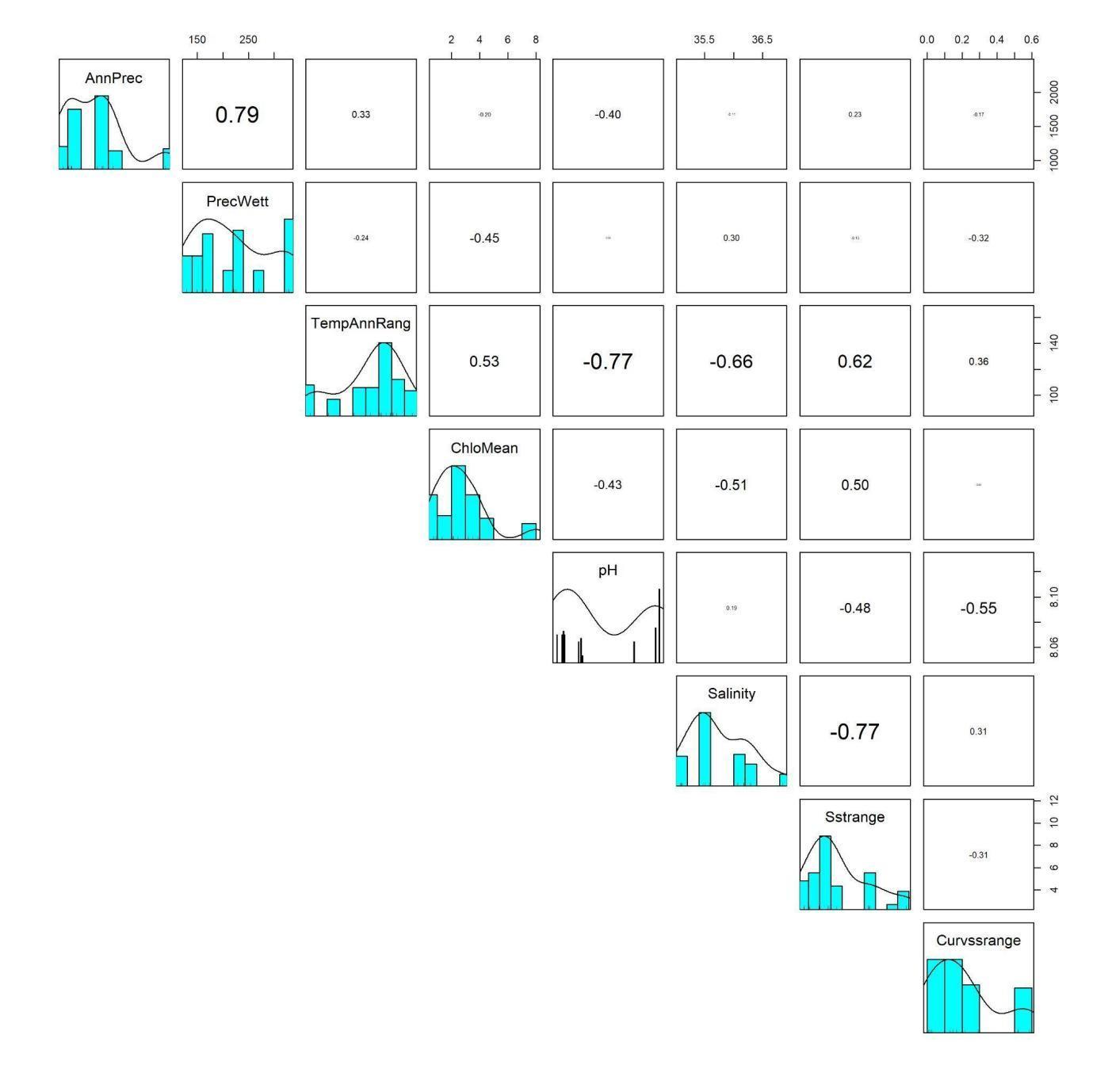


## Fig. S2. Correlation coefficients between each pair of environmental predictors used for the association tests (RDA and LFMM) of *Littoraria flava*’s populations, with their font size scaled to their |r| on the upper diagonal. All the coefficients were obtained with the pairs.panels function from psych R package. The diagonal shows histograms of the correlation between the pair of predictors.


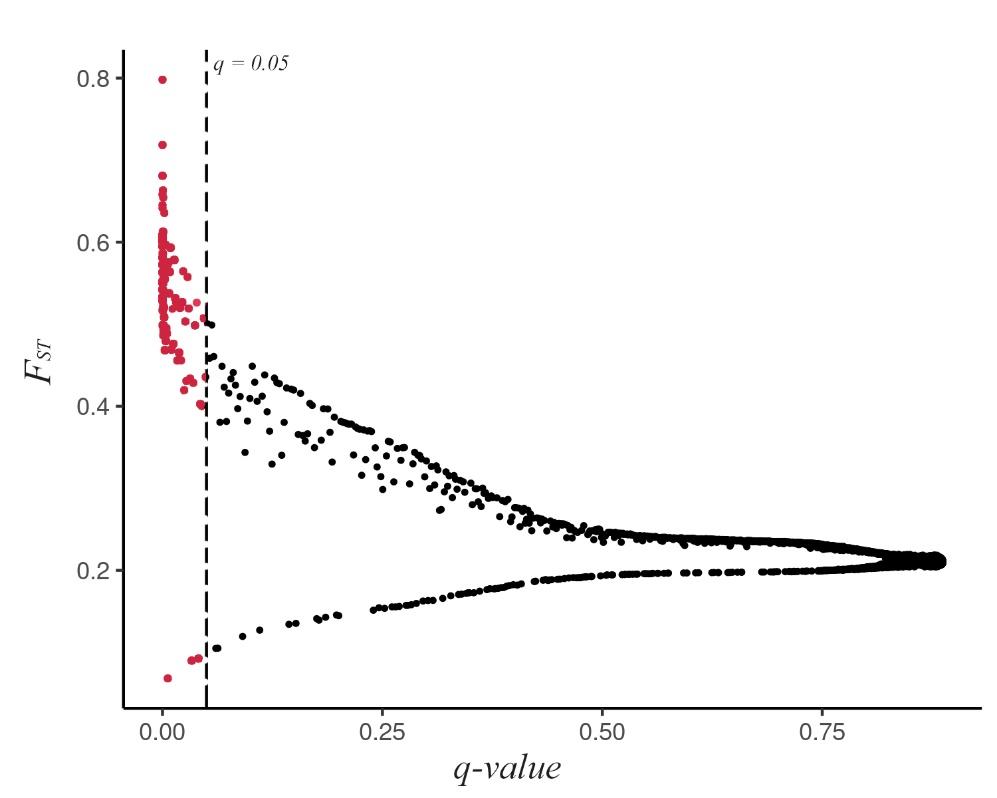


## Fig. S3. Genomic scan from BayeScan performed for individual GBS derived SNPs of *Littoraria flava*. Each dot represents a single SNP with their respective q-value on the x-axis and *F_ST_* on the y-axis. The black dashed line indicates the adopted significance level (*q* < 0.05) and therefore SNPs in red at the left of the vertical line represent outliers.


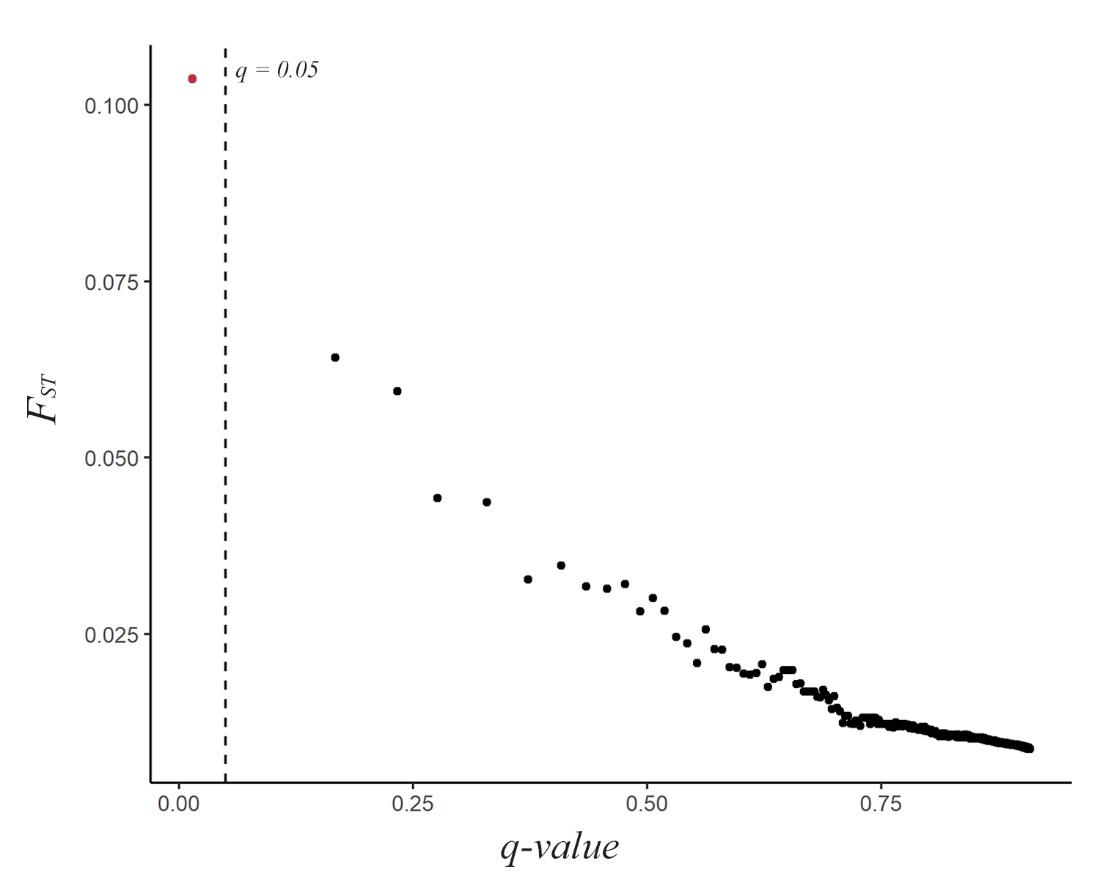


## Fig. S4. Genomic scan from BayeScan performed for individual RNA-Seq derived SNPs of *Littoraria flava*. Each dot represents a single SNP with their respective q-value on the x-axis and *F_ST_* on the y-axis. The black dashed line indicates the adopted significance level (*q* < 0.05) and therefore the SNP in red at the left of the vertical line represents an outlier.


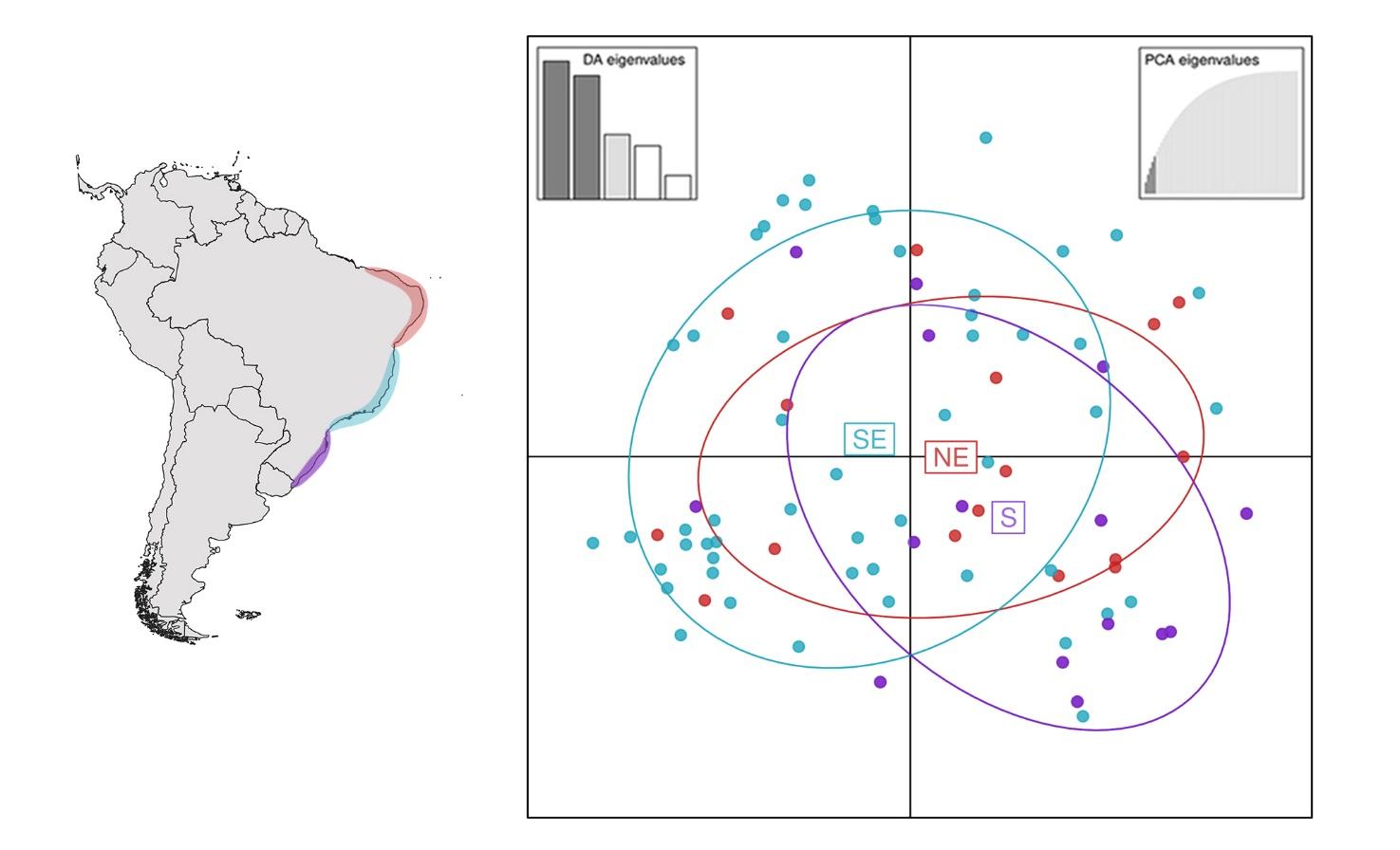


## Fig. S5. Discriminant analysis of principal components (DAPC) performed based on 69 GBS candidate SNPs from 85 individuals of *Littoraria flava*. The distribution of individuals across the two first principal components (PCs) colored according to regions from Brazilian coastline, which extensions can be visualized on the map at the left. Each dot represents an individual. The DAPC was performed using 5 PCs, as suggested by the α-score optimization.


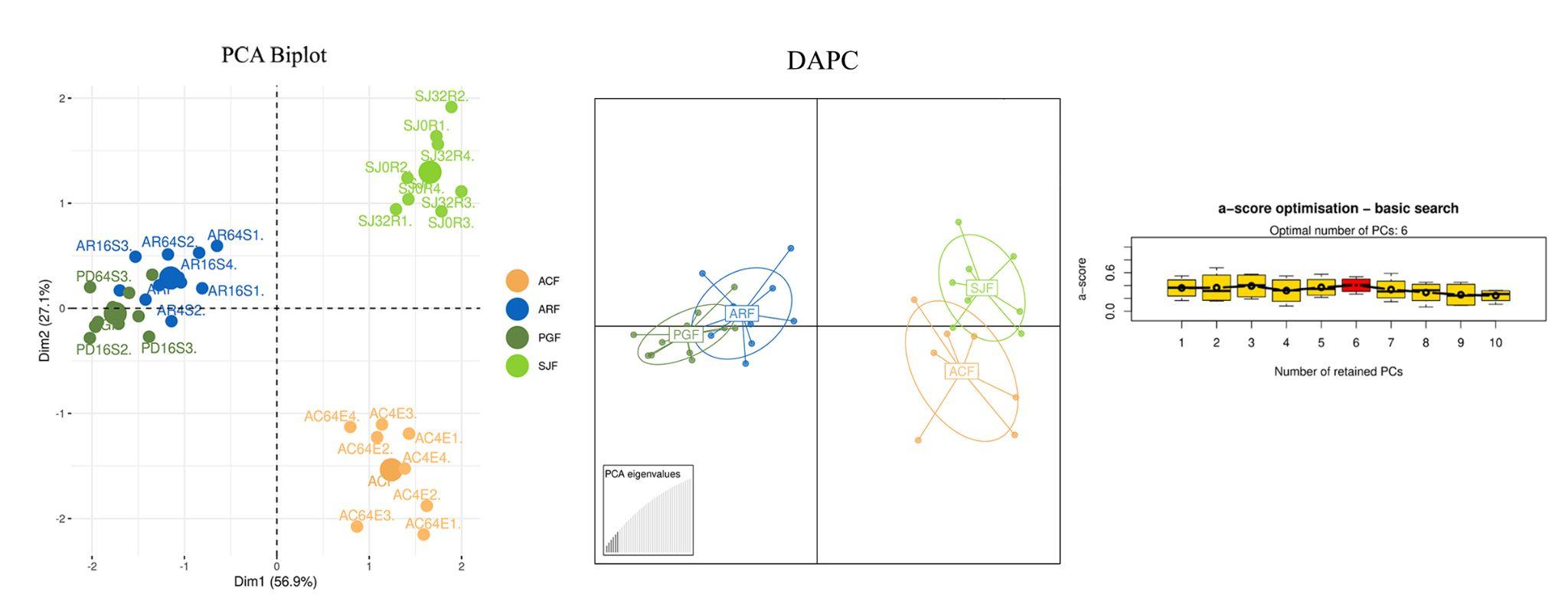


## Fig. S6. Multivariate analysis based on 506 putative adaptive SNPs derived from transcriptome of *Littoraria flava*. Principal Component Analysis (PCA, left) and Discriminant Analysis of Principal Components (DAPC, center) based on the mean frequency of SNPs indicates a clear segregation of local populations, indicated by different colors. Abbreviations as in Table S1. The α-score optimization (right) indicates the optimum number of principal components (PCs) for the analysis.

# Appendices S1-S4

<https://drive.google.com/drive/u/1/folders/1epROnDGIWyF3RSfo-Gqt04sjtOfBcABW>
